# Health Tract, No 65

**Published:** 1865

**Authors:** 


### HEALTH TRACT, No 65.

### PHYSIOLOGICAL APHORISMS.

1. **The** foundation of three fourths of all cases of consumption is laid before the age of twenty-five years; in women, during their teens.

2. The hereditary element is not of special account as a cause of consumption, as less than twenty-five per cent of cases are clearly of consumptive parentage.

3. One of the ruling causes of disease and premature death, in large cities, is found in that exhausting strain of the mental energies in the struggle for subsistence—a death-race for bread.

4. Insanity runs in families; but, as in the case of family likeness, it sometimes overleaps a generation or more.

5. Personal resemblance entails like characteristics of mind and disposition.

6. A current of the purest air from the poles, for half an hour, on a person sleeping, sitting still, or overheated, is a thousand-fold more destructive of health and fatal to life than the noisomeness of a crowded room or vehicle, or the stench of a pig-stye for thrice the time.

7. To exercise in weariness, increased by every step, is not only not beneficial, it is useless and worse than useless; it is positively destructive.

8. As no good traveler, after having fed his horse, renews his journey in a trot, but with a slow walk, gradually increasing his pace, so in getting up to address an assembly for a continued effort, the first few sentences should be uttered in a low, slow tone, gradually intensified, otherwise the voice will break down in a very few minutes, with coughing or hoarseness.

9. A growing inability to sleep in sickness is ominous of a fatal result; in apparent health, it indicates the failure of the mind and madness; so, on the other hand, in disease or dementia, a very slight improvement in the sleeping should be hailed as the harbinger of restoration.

10. No one can possibly sink if the head is thrust entirely under water, and in this position a novice can swim as easily as walk, and get to shore readily by lifting the head at intervals, for breath.

11. Intense thirst is satiated by wading in water, or by keeping the clothing saturated with water, even if it is taken from the sea.

12. Water can not satisfy the thirst which attends cholera, dysentery, diarrhea, and some other forms of disease; in fact, drinking cold water seems to increase the thirst, and induce other disagreeable sensations; but this thirst will be perfectly and pleasantly subdued, by eating a comparatively small amount of ice, swallowing it in as large pieces as practicable, and as much as is wanted.

13. Inflammations are more safely and far more agreeably subdued by the application of warm water than of cold.

14. Very excessive effort in a short space of time, as in running, or jumping a rope, etc., has repeatedly caused instant death, by apoplexy of the lungs, the exercise sending the blood there faster than it can be forwarded to the heart, and faster than it can be purified by the more infrequent breathing on such occasions.

15. No disease ever comes without a cause or without a warning; hence endeavor to think back for the cause, with a view to avoid it in future, and on the instant of any unpleasant bodily sensation, cease eating absolutely until it has entirely disappeared, at least for twenty-four hours; if still remaining, consult a physician.

16. The more clothes a man wears, the more bed-covering he uses, the closer he keeps his chamber, whether warm or cold, the more he confines himself to the house, the more numerous and warm his night-garments, the more readily will he take cold, under all circumstances, as the more a thriftless youth is helped, the less able does he become to help himself.

